# *Bacteroides thetaiotaomicron* Ameliorates Experimental Allergic Airway Inflammation *via* Activation of ICOS^+^Tregs and Inhibition of Th2 Response

**DOI:** 10.3389/fimmu.2021.620943

**Published:** 2021-03-17

**Authors:** Wenhui Pang, Yan Jiang, Aifeng Li, Jisheng Zhang, Min Chen, Li Hu, Zhiyuan Li, Dehui Wang

**Affiliations:** ^1^ Department of Otolaryngology-Head and Neck Surgery, The Affiliated Hospital of Qingdao University, Qingdao, China; ^2^ ENT Institute and Department of Otorhinolaryngology, Eye & ENT Hospital, Fudan University, Shanghai, China; ^3^ Medical Research Center, Key Laboratory of Otolaryngology-Head and Neck Surgery, The Affiliated Hospital of Qingdao University, Qingdao, China

**Keywords:** allergic airway diseases, 16s rRNA gene, dysbiosis, *Bacteroides thetaiotaomicron*, regulatory T cells

## Abstract

Inhibition of allergic airway diseases (AAD) by immunomodulation of the adaptive immune system through restoration of the enteric dysbiosis is an emerging therapeutic strategy. Patients with allergic rhinitis (n = 6) and healthy controls (n = 6) were enrolled, and gut microbiome composition analysis was performed by 16S rDNA sequencing. We also established an ovalbumin (OVA)-induced allergic airway inflammation murine model. Dysbiosis of the gut flora was observed in both AAD patients and the mice, with the decrease of the biodiversity and the quantity of the Bacteroidetes phylum. Oral application of *Bacteroides (B.) thetaiotaomicron* ameliorated the symptoms of OVA-induced airway hyperresponsiveness (AHR) and attenuated the airway inflammation in mice. In addition, nasal lavage fluid (NALF) and bronchoalveolar lavage fluid (BALF) from AAD mice orally administered with *B. thetaiotaomicron* showed reduced numbers of immune cells, and diminished secretion of T helper (Th)-2 cytokines (IL-4, IL-5, and IL-13) compared with the corresponding control mice, whereas the levels of Th1 cytokineIFN-γ was not changed in both the groups. When *B. thetaiotaomicron* was co-administered with metronidazole in AAD mice, the immunomodulatory effect was weakened and the allergic inflammatory response was aggravated. The ratios of CD4^+^Foxp3^+^ cells, CD4^+^ICOS^+^ T cells, CD4^+^ICOS^+^ Foxp3^+^ regulatory T cells, and IL-10-expressing CD4^+^Foxp3^+^ cells were increased in lymphocytes of spleen, mesenteric, and cervical lymph nodes of AAD mice administrated with *B. thetaiotaomicron*. Therefore, our data indicate that oral administration of *B. thetaiotaomicron* effectively inhibited the development of AAD in murine model; inhibition was mediated by the activation of Tregs and inhibition of Th2 response without promoting a Th1 response.

## Introduction

Allergic rhinitis and asthma are different manifestations of allergic airway disease (AAD) in upper and lower respiratory tracts, having similar pathogenesis and clinical characteristics (1). As per epidemiological investigations, in recent years the incidence of respiratory allergic inflammatory diseases has significantly increased with the change in living environment in many regions or countries including China. At present, there are at least 300 million asthma patients in the world, and the incidence of allergic rhinitis is as high as 20% (2, 3). However, specific underlying causes of this disease are still unknown.

AAD is immunologically dominated by the T-helper type 2 (Th2) cells in the respiratory tract (1). A re-exposure to specific antigens such as pollen and dust mites, stimulates IgE production by the plasma cells and activates the sensitized mast cells degranulation. This results in the release of inflammatory mediators, such as leukotrienes, histamine, and prostaglandins, inducing T-helper type 1 (Th1)/Th2 immune-imbalance. Th2 cells assemble and release a large amount of IL-4, IL-5, IL-13, and other inflammatory cytokines, causing the infiltration of eosinophils in the respiratory tract mucosa to evoke the clinical symptoms of allergy (1, 4).

Th2 polarization and abnormal levels of IgE mirror the dysfunctional immune regulatory system in AAD patients. The regulatory T cells (Tregs), regulatory B cells (Bregs), tolerogenic dendritic cells (DCs), and immuneregulatory cytokines such as transforming growth factor (TGF)-β and interleukin (IL)-10 comprise the immune regulatory system (5–7). Inducible co-stimulatory molecule (ICOS) (CD278), a type of co-stimulatory molecule, is expressed by activated T cells and Tregs (8). ICOS enhances the proliferation, function, and survival of Tregs and plays an important role in a variety of autoimmune diseases and allergic diseases especially asthma (9, 10).

Adequate microbial stimuli from commensal microbiota are indispensable for the maintenance of immunefunctions in the body. Dysbiosis disturbs the immune system, potentially leading to inflammation. Studies have associated early dysbiosis of the gut microbiome with allergies (11, 12). A pivotal study reported by Noverr et al. demonstrated that allergies can develop as a consequence of an altered gut microbiota, suggesting that alterations in the gut microbiota can facilitate an immunological state that is predisposed to respiratory allergies (13). In addition, Vital et al. found that a locally induced pulmonary allergic response is affecting the composition of the intestinal microbiome, indicating bidirectional gut-lung communications (14). One of the important strategies of AAD inhibition is immunomodulation of the adaptive immune system through restoration of enteric dysbiosis (15). In the present study, we investigated the fecal microbiome of AAD based on 16S rDNA sequencing and found a significantly lower abundance of the Bacteroidetes phylum in both AAD patients and mice. *B. thetaiotaomicron* is one of the most abundant bacteria in gut flora, which belongs to the genus Bacteroides. We were specifically interested in exploring the potential immunomodulatory effect of orally administered *B. thetaiotaomicron* on treating AAD.

## Materials and Methods

### Patients and Sample Collection

The study guidelines were approved by the Affiliated Hospital of Qingdao University Review Board and the Affiliated Eye, Ear, Nose, and Throat Hospital of Fudan University Review Board (IRB No. QYFYWZLL26053). Patients aged from 18 to 65 years were recruited from February 2013 to April 2014 and categorized into allergic rhinitis (n = 6) or healthy control (n = 6) groups. Fresh stools were collected and immediately stored at −80°C until further use. Prior to sampling none of the patients received antibiotics for at least 1 month. Pregnant and lactating women and patients with organic heart disease, asthma, or abnormal liver or kidney functions were excluded.

### Animals

Female BALB/c (5 to 6 weeks) were purchased from Beijing Vital River Laboratory Animal Technologies Co. Ltd (Beijing, China) and maintained under specific-pathogen-free (SPF) conditions. The animal experiment procedures were approved by the Animal Care Committee of Qingdao University and Fudan University (IACUC No. AHQU20170831).

### OVA Sensitization and Challenge

Mice were intraperitoneally (i.p.) injected with 200 μl PBS or 40 μg OVA (Sigma) in 2 mg/100 μl Al(OH)_3_ gel (Thermo) suspended in PBS on days 1, 3, 5, 7, 9, 11, and 13 (sensitization). This was followed by topical application of 20 μl PBS or 5% OVA solution once daily *via* nasal drops from day 21 to 27 (challenge). Mouse feces were collected 24h after challenge.

### 16S rRNA Gene Amplification and Sequencing

Extraction of bacterial DNA from 0.5g of human or mouse feces was performed by a FastDNA Spin Kit (OMEGA) following the manufacturer’s instructions. The variable region V3–V4 was amplified by polymerase chain reaction (PCR) using bacteria/archaeal primers 341F/805R with barcodes. The amplified DNA was quantified using a Qubit 2.0 DNA detection kit (Thermo Scientific). 10 ng of DNA was taken from each sample, and the final sequencing concentration was 20 pmol. Microbial metagenomic 16S rDNA sequencing was performed.

### *B. thetaiotaomicron* Treatment

**Figure 1:**
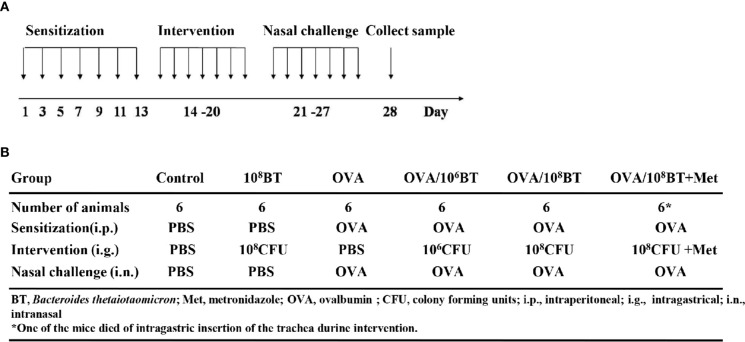
The experimental protocol **(A)** and different groups **(B)**. Mice were given *B. thetaiotaomicron* daily from Day 14–20, with OVA-sensitization on Day 1, 3, 5, 7, 9, 11, and 13, and nasal challenge with 5% OVA from Day 21–27. Then mice were sacrificed on Day 28 to collect tissues or samples for experimental analysis. Mice were divided into six different groups in this work. Each experimental group consisted of six mice.

The *B. thetaiotaomicron* strain 29148 was obtained from ATCC (USA). Prior to oral administration of bacteria in mice, the concentration of the bacteria was adjusted to 10^6^ or 10^8^ colony forming units (CFUs)/ml with PBS. After OVA sensitizing, PBS, *B. thetaiotaomicron* (10^6^ CFU/ml, 10^8^ CFU/ml), or *B. thetaiotaomicron* (10^8^ CFU/ml) combined with 0.5 mg metronidazole was administered by oral gavage from day 14 to 20 once per day, respectively (**Figure 1**).

### Observation of Nasal Symptoms

Sneeze numbers were counted by reviewing the videos of daily activities of the animals in an observation cage. Counting was immediately performed for a period of 10 min after the final intranasal challenge.

### Cell Assays for NALF and BALF

After partial tracheal resection, the nasal and lungs were perfused three times with 0.5 ml PBS, and NALF and BALF samples were collected 24 h after the last OVA challenge. The samples were centrifuged at 3,000 rpm for 5 min, and the lavage supernatant was collected for enzyme-linked immunosorbent assay (ELISA). The cell pellets were re-suspended in 0.1 ml PBS, and the cells were counted using a hemocytometer. Cytospin preparations and Wright-Giemsa staining were then performed for different cell counts followed by counting the absolute numbers of each cell type under a light microscope (Leica).

### ELISA

Mouse serum samples were prepared 24 h after the last OVA challenge, and OVA-specific IgE and IgG1 levels were measured using commercially available ELISA kits (Chondrex). Concentrations of cytokines IFN-γ, IL-4, IL-5, IL-10, and IL-13 in both NALF and BALF samples were assessed by ELISA as per the manufacturer’s instructions (Merck Millipore).

### Histopathological Analysis

The nasal and lung were excised 24 h after the final challenge and fixed in 10% formalin. The fixed nasal was further decalcified with 10% EDTA for 7 days. The samples were embedded in paraffin, and 4-μm sections were cut and fixed on the glass slides. The slides were deparaffinized and stained with HE or toluidine blue. For inflammation scoring of the lungs, a reproducible scoring system was used as previously described (16). Five fields per sample were carefully examined for calculating number of eosinophils or mast cells in the nasal mucosa and lungs.

### Fluorescence-Activated Cell Sorting (FACS) Analysis

Mesenteric lymph nodes (LNs), cervical LNs and spleen cells were collected 24 h after the final challenge. These cells were then washed with 1 × PBS and incubated with anti-CD16/CD32 mAb (eBioscience) for 15 min to block nonspecific Ab binding. Cells were subsequently stained with PercpCy5.5–anti-CD25 (eBioscience), FITC–anti-CD4 (eBioscience), and PE-anti-ICOS (eBioscience) at 4°C for 30 min. The cells were fixed and permeabilized for 30 min after washing. APC-anti-Foxp3 mAb (eBioscience) was added to the cells, and the cells incubated for another 30 min at 4°C. Data were recorded by a FACS Calibur flow cytometer (BD Biosciences) and analyzed using FlowJo software. For intracellular cytokine staining of IL-10, cells isolated from mesenteric LNs, cervical LNs and spleens were stimulated for 4h with ionomycin (250 ng/ml) and PMA (10 ng/ml) in RPMI 1640 medium containing 10% FBS, 1% streptomycin and penicillin, 2mM L-glutamine and 10mM HEPEs in 5% CO_2_ at 37°C. Then the cells were stained with PercpCy5.5–anti-CD25 (eBioscience), FITC–anti-CD4 (eBioscience), APC-anti-Foxp3 (eBioscience), and PE-anti-IL-10 (eBioscience).

### Statistical Analysis

Data were presented as mean ± SD. All data analyses were performed with SPSS20.0 and GraphPad Prism 6.0. For multiple comparisons of data between more than two groups, we used one-way analysis of variance (ANOVA) followed by Tukey’s post-hoc test. A p-value < 0.05 was considered statistically significant.

## Results

### Bacteroides Are Reduced in Allergic Rhinitis Patients and Mice

**Figure 2:**
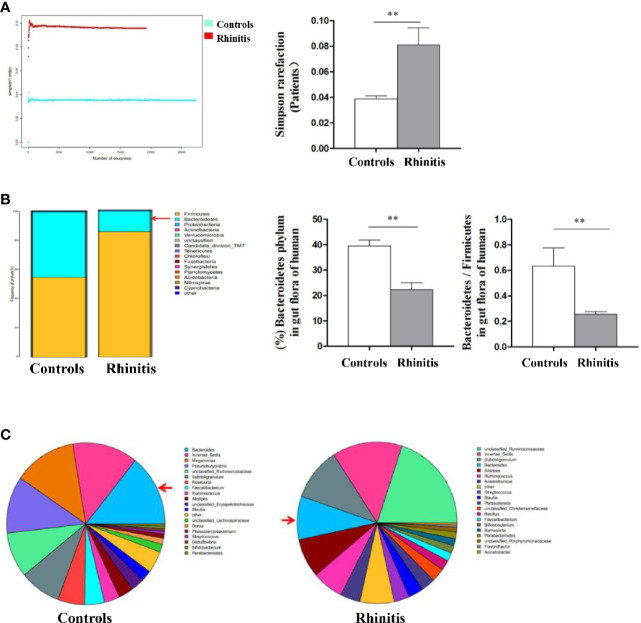
The diversity and abundance of intestinal microflora in patients with allergic rhinitis. **(A)** The Simpson rarefaction in allergic rhinitis patients increased compared with that in healthy controls. **(B)** Bacterial composition and abundance at the phylum level. Red arrow: Bacteroidetes. **(C)** Bacterial composition and abundance at the genus level. Red arrow: Bacteroides. Bar graphs represent mean ± SD (n = 6). **P < 0.01.

**Figure 3:**
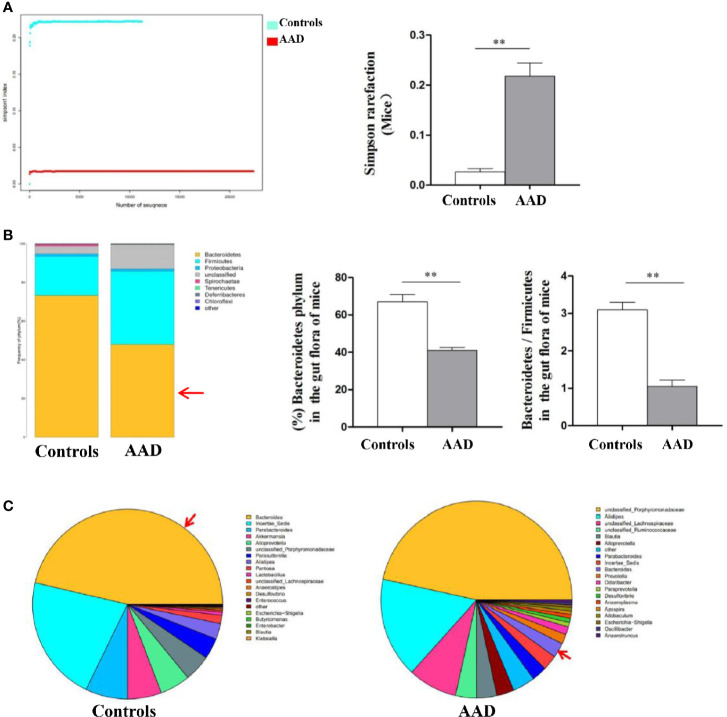
The diversity and abundance of intestinal microflora in murine model of OVA-induced allergic airway inflammation. **(A)** The Simpson rarefaction in AAD mice increased. **(B)** Bacterial composition and abundance at the phylum level. Red arrow: Bacteroidetes. **(C)** Bacterial composition and abundance at the genus level. Red arrow: Bacteroides. Bar graphs represent mean ± SD (n = 6). **P < 0.01.

Stool specimens collected from patients with allergic rhinitis and healthy controls. 16S rDNA technology was used to detect the diversity and abundance of intestinal microflora in patients with allergic rhinitis. We found that the Simpson rarefaction in allergic rhinitis patients significantly increased as compared with that in healthy controls, indicating an expected decrease of biodiversity in allergic rhinitis patients (**Figure 2A**). The bacterial composition and abundance at the phylum and genus levels are shown in **Figures 2B** and **C** respectively. In healthy controls, taxonomic classification suggested a high abundance of phylum Firmicutes followed by phylum Bacteroidetes, while an expected lower abundance of Bacteroidetes was found in allergic rhinitis patients at phylum level (**Figure 2B**). At the genus level, abundance of Bacteroides in patients with rhinitis was significantly less as compared with that in healthy controls (**Figure 2C**). We obtained similar results including decreased biodiversity (**Figure 3A**), lower abundance of phylum Bacteroidetes (**Figure 3B**) and Bacteroides (**Figure 3C**) in the gut microflora of a murine model of OVA-induced allergic airway inflammation. Taken together, enteric dysbiosis with the decrease of abundance of Bacteroides existed in allergic rhinitis patients and mice.

### *B. thetaiotaomicron* Ameliorates AHR

**Figure 4:**
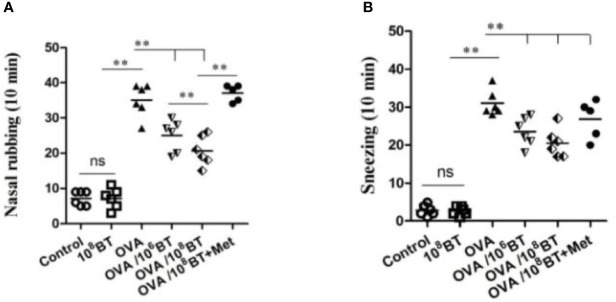
The changes of allergic symptoms. OVA induced an obvious AHR in mice with increased frequency of nasal rubbing **(A)** and sneezing **(B)**. The occurrences of nasal rubbing and sneezing were noticeably reduced in OVA/BT group, while OVA/BT+Met inhibited the effect in ameliorating allergic symptoms. **P < 0.01.

*B. thetaiotaomicron* is one of the most abundant bacteria in gut flora, which belongs to the genus Bacteroides. We further tested *B. thetaiotaomicron* in OVA-induced allergic airway inflammation murine model system to explore the potential role of this bacteria in treating allergic airway inflammation (**Figure 1**). Firstly we measured the frequency of allergic symptoms. AHR was induced in mice of OVA group, resulting in an increased frequency of nasal rubbing (**Figure 4A**) and sneezing (**Figure 4B**). Interestingly, in the OVA/10^6^BT and OVA/10^8^BT groups the occurrences of nasal rubbing and sneezing were lower as compared with the occurrences in the OVA group; in the OVA/10^8^BT+Met group metronidazole inhibited the effect in ameliorating allergic symptoms. The allergic symptoms between control and 10^8^BT groups had no noteworthy differences (**Figure 4**).

### *B. thetaiotaomicron* Attenuates OVA–Induced Airway Inflammation in Mice

**Figure 5:**
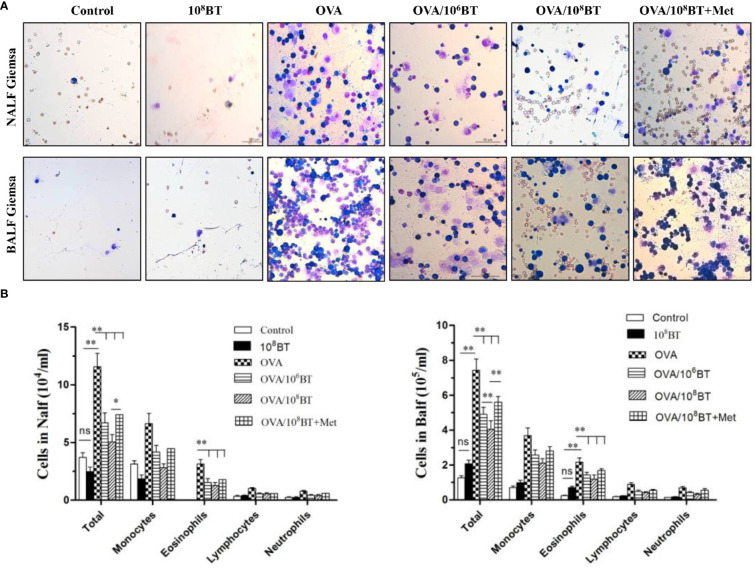
OVA/BT inhibited OVA-induced airway inflammation. **(A)** Wright-Giemsa staining of inflammation cells in NALF and BALF at 24 h after final challenge. **(B)** Total inflammation cells and eosinophils in NALF and BALF. Bar graphs represent mean ± SD.*P < 0.05, **P < 0.01. ns, no significance.

We further examined the inflammatory cells from NALF and BALF samples (**Figure 5**). More inflammatory cells—eosinophils, neutrophils, monocytes, and lymphocytes—were detected in OVA group than the control group. Oral *B. thetaiotaomicron* administration before challenge in the OVA/10^6^BT and OVA/10^8^BT groups significantly reduced total cells and eosinophils in NALF and BALF. We also evaluated the mast cell and eosinophil infiltration in the nasal and lung by histological analysis to investigate the effect of oral *B. thetaiotaomicron* before challenge. There were no changes between the control and 10^8^BT groups, while OVA group had mast cell (**Figure S1**) and eosinophil infiltration (**Figure S2**) in the nasal and lung. In the OVA/10^6^BT and OVA/10^8^BT groups, we found a significant suppression of mast cell and eosinophil infiltration by oral *B. thetaiotaomicron*. When metronidazole was co-administered with *B. thetaiotaomicron*, the suppression effect was weakened, and the airway inflammation was aggravated in OVA/10^8^BT + Met group. These findings suggest that oral administration of *B. thetaiotaomicron* attenuates OVA-induced airway inflammation.

### *B. thetaiotaomicron* Inhibits OVA-Induced Airway Th2 Cytokines and Serum OVA-Specific IgE

**Figure 6:**
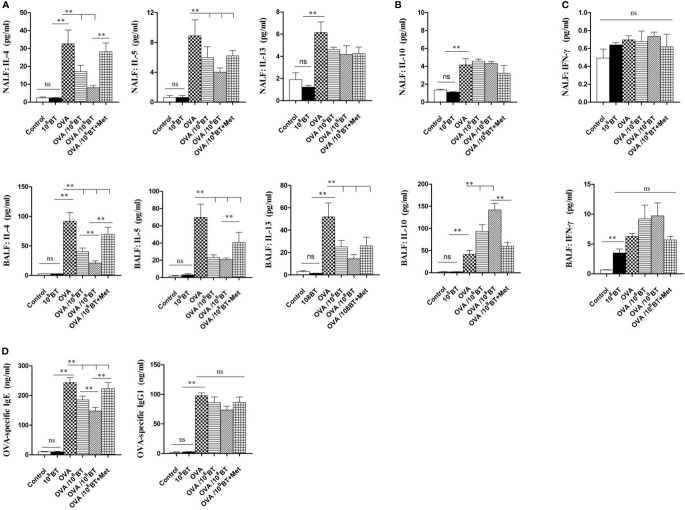
The changes of cytokines IL-4, IL-5, IL-13 **(A)**, IL-10 **(B)** and IFN-γ **(C)** in NALF and BALF and serum OVA-specific IgE and IgG1 levels **(D)**. Oral *B. thetaiotaomicron* application inhibited secretion of Th2 cytokines IL-4, IL-5 and IL-13 and increased levels of IL-10 in BALF. Levels of Th1 cytokine IFN-γ in the lavage liquid were not significantly changed. Oral *B. thetaiotaomicron* application also reduced serum OVA-specific IgE production induced by OVA challenge significantly, but not IgG1 production.**P < 0.01. ns, no significance.

Levels of Th2 cytokines (IL-4, IL-5, and IL-13) in NALF and BALF samples of OVA group were significantly higher as compared with that of control group; OVA/10^6^BT and OVA/10^8^BT groups showed relatively lower Th2 cytokine levels as compared with OVA group (**Figure 6A**). When *B. thetaiotaomicron* was co-administered with metronidazole (OVA/10^8^BT + Met group), the inhibitory effect of *B. thetaiotaomicron* on Th2 cytokine levels was weakened. However in BALF samples, levels of IL-10, the main cytokine secreted by regulatory T cells to mediate immune suppression, were significantly higher in OVA/10^6^BT and OVA/10^8^BT groups than OVA model group (**Figure 6B**). Also, levels of Th1 cytokine IFN-γ, were not significantly changed among the OVA, OVA/10^6^BT, OVA/10^8^BT, and OVA/10^8^BT + Met groups in the lavage liquid (**Figure 6C**). Furthermore, oral administration of *B. thetaiotaomicron* in the OVA/10^6^BT and OVA/10^8^BT groups significantly reduced the production of OVA-induced serum IgE but not that of IgG1 (**Figure 6D**). The co-administration of metronidazole in OVA/10^8^BT+Met group impaired the inhibition of IgE induced by *B. thetaiotaomicron*, similar to the findings of Th2 cytokines.

### *B. thetaiotaomicron* Increases ICOS and IL-10 Expression in Treg Cells in the Mesenteric and Cervical Lymph Nodes (LNs) and Spleen

**Figure 7:**
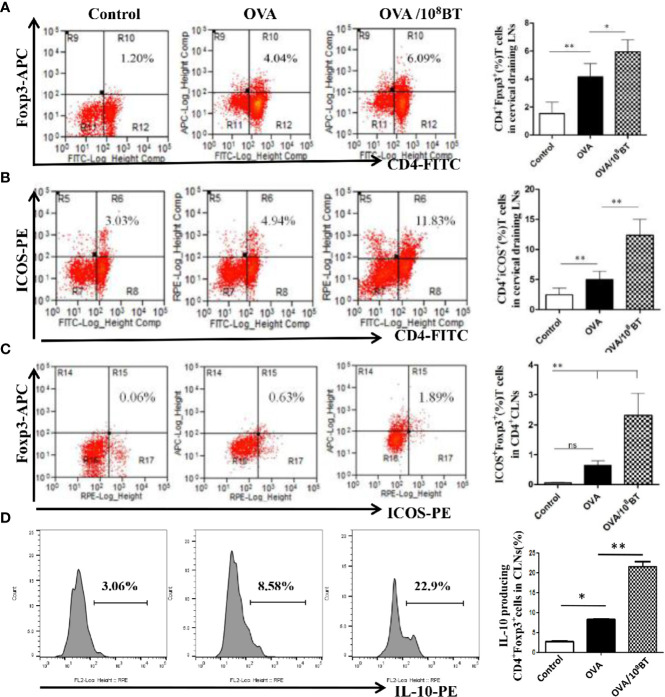
*B. thetaiotaomicron* induced ICOS expression on Tregs and amplification of IL-10-expressing CD4^+^Foxp3^+^Tregs in draining cervical LNs. Representative scatter plots and ration of the fraction of CD4^+^Foxp3^+^ cells **(A)**, CD4^+^ICOS^+^T cells **(B)** and CD4^+^ICOS^+^Foxp3^+^ regulatory T cells **(C)**. Representative histogram showing expression of IL-10 in CD4^+^Foxp3^+^ Tregs **(D)**. Bar graphs represent mean ± SD. n = 6, *P < 0.05, **P < 0.01. ns, no significance.

ICOS enhances the proliferation, survival, and function of Treg cells and has critical immunoregulatory implications (9). We investigated the effects of oral administration of *B. thetaiotaomicron* on the regulation of ICOS and Tregs in spleen, mesenteric LNs and cervical LNs that drains the nasal mucosa and further examined the frequency of CD4^+^ T cells expressing either CD25 or ICOS, and Foxp3, as well as intracellular cytokine staining for IL-10 at specific time intervals. Ratios of CD4^+^Foxp3^+^ cells and CD4^+^ICOS^+^ cells were slightly increased in lymphocytes of the OVA model group as compared with that of the control group. When *B. thetaiotaomicron* was administered, ratios of CD4^+^Foxp3^+^ cells, CD4^+^ICOS^+^T cells, and CD4^+^ICOS^+^Foxp3^+^ regulatory T cells were obviously upregulated in the lymphocytes of the spleen (**Figure S3**), mesenteric LNs (**Figure S4**) and cervical LNs (**Figure 7**) of OVA/10^8^BT group as compared with that of the OVA model group. In addition, *B. thetaiotaomicron* increased ratio of IL-10-expressing CD4^+^Foxp3^+^ cells in the spleen (**Figure S3D**), mesenteric LNs (**Figure S4D**) and cervical LNs (**Figure 7D**) relative to OVA model group. These data indicated that application of *B. thetaiotaomicron* before allergen challenge was capable of establishing adequate Tregs to regulate immune system for preventing AHR *via* secretion of IL-10.

## Discussion

The healthy gut microbiota is a stable, diverse, resilient, and resistant microbial ecosystem. Firmicutes and Bacteroidetes are two major phyla, together representing ∼90% of the gut microbiota. Gut microbiota have a significant effect on systemic immunity and metabolism, which are involved in the occurrence and development of AAD (17–19). In children with allergic rhinitis and asthma, the abundance of phylum Firmicutes was lower as compared with that in healthy controls (20). However, we observed enteric dysbiosis with the decrease in biodiversity and lower abundance of phylum Bacteroidetes in both allergic rhinitis adult patients and mice. A plausible explanation for this variation can be that the gut microbiota coexists with the human body and changes with age. In the first 2–3 years of life, the gut microbiota varies extensively in composition and metabolic functions. After this period, the gut microbiota demonstrates adult-like more stable and diverse microbial species. However, in old age, the gut microbiota alters drastically and shows less diversity compared to younger age, which promotes various gut-related diseases. The number of gram negative bacteria increases during aging, which secretes lipopolysaccharide and causes inflammation in human gut (21, 22). A decrease of the Bacteroidetes to Firmicutes ratio was also observed in old compared to young mice (14).

*B. thetaiotaomicron* is one of the most efficient polysaccharide-degrading bacteria and contains exclusive polysaccharide-degrading enzymes not found in humans. *B. thetaiotaomicron* can produce up to 260 kinds of enzymes that can decompose starch, glycogen and cellulose into small molecules of glucose, thus helping the human body to effectively extract nutrients from wheat germ, vegetables, fruits and other foods. *B. thetaiotaomicron* can quickly adjust more than 25% of its genes to the active state according to the changes of food sources, so as to digest new nutrients and maintain the health of the whole intestinal flora (23–25). The *B. thetaiotaomicron* ATCC 29148 strain is a standard strain originally purified from the feces of healthy adults (25). In the present study, we performed intervention assays using *B. thetaiotaomicron* ATCC 29148 (10^6^CFUs and 10^8^CFUs) and OVA-induced allergic airway inflammation murine model. The oral administration of ATCC 29148 in mice ameliorated the allergic symptoms of nose and reduced the frequency of scratching and sneezing. It also inhibited eosinophilic inflammation of nasal mucosa and bronchoalveolar tissues and the mast cell infiltration. The exudation of inflammatory cells, especially eosinophils, in the nasal cavity and alveolar lavage solution was also reduced. In addition, the expression levels of Th2 cytokines (IL-4, IL-5, and IL-13) and OVA-specific IgE were down-regulated. IL-4, IL-5, and IL-13 are known to regulate the growth and differentiation of eosinophils (26). Oral probiotics can alter the respiratory microbiota and have been advocated as a novel therapeutic strategy for AAD (27). Our findings are consistent with other reports demonstrating improvement in allergic rhinitis and asthma inflammation through oral administration of *Bifidobacterium breve* (28) and *Lactobacillus plantarum* (29). However, levels of the main cytokine secreted by Tregs to mediate immune suppression, IL-10, were significantly higher in ATCC 29148 intervention group as compared with the levels in control group. In addition, the level of Th1 cytokine, IFN-γ, was not influenced by *B. thetaiotaomicron* ATCC 29148, suggesting that ATCC 29148 influences Th1/Th2 equilibrium mainly through the inhibition of Th2 response without promoting a Th1 response. These observations can be explained by the characteristic differences between *B. thetaiotaomicron* and other probiotics. There are characteristic differences in growth, carbohydrate consumption or metabolite production among various species of bacteria. The immune responses induced by various species of bacteria are different. It has been shown that *Bifidobacterium adolescentis* did not challenge any production of bacteria specific serum antibodies in comparison to germ-free rat, while *B. thetaiotaomicron* alone can induce a humoral response characterized by IgA and IgG production (30).

Interestingly, immunomodulatory effect of *B. thetaiotaomicron* ATCC 29148 was weakened and the allergic inflammatory response was aggravated, when ATCC 29148 was orally administered in mice in combination with metronidazole. These findings suggest that metronidazole can block the immunomodulatory effect of *B. thetaiotaomicron*. The overuse of antibiotics may result in strong selection pressure and influence the inherent flora of human intestinal tract, which is one of the reasons for the rapid increase of AAD incidence in recent years (31). McKeever et al. confirmed that the incidence of AAD is closely related with the use of antibiotics in infants (32). Therefore, antibiotics should only be used when needed, and their use should be regulated.

Tregs play an indispensable role in maintaining immune homeostasis and contribute to allergic disease management by suppressing Th2-type immune responses. The balance between Th2 and Tregs is crucial for the development or suppression of allergic airway inflammation (33). ICOS is expressed on antigen-primed T cells—activated effector T cells, Th2 cells, memory T cells, and Tregs—and plays a key role in T cell activation and differentiation (8, 9). Here, we analyzed the expression of ICOS on CD4^+^ T cells and Foxp3^+^ Tregs to investigate the immunomodulatory effect of *B. thetaiotaomicron* on allergic airway inflammation in mice. After oral administration of *B. thetaiotaomicron*, ratios of CD4^+^Foxp3^+^ cells, CD4^+^ICOS^+^ T cells, and CD4^+^ICOS^+^Foxp3^+^ regulatory T cells were higher in the lymphocytes of spleen, mesenteric and cervical LNs of OVA/10^8^BT groups compared with that of the OVA model group. *B. thetaiotaomicron* also increased ratio of IL-10-expressing CD4^+^Foxp3^+^ cells in the spleen, mesenteric LNs, and cervical LNs relative to OVA model group. These observations suggest that *B. thetaiotaomicron* intervention can induce the expansion of CD4^+^ICOS^+^Foxp3^+^ and IL-10^+^CD4^+^Foxp3^+^regulatory T cells, exerting an immunosuppressive effect on Th2 type response.

Collectively, our study demonstrated that enteric dysbiosis with the decrease of abundance of Bacteroides existed in AAD; oral administration of *B. thetaiotaomicron* significantly attenuates allergic airway inflammation in OVA-induced AAD model. *B. thetaiotaomicron* may open up novel possibilities in terms of therapeutic interventions for AAD. Alteration of the ratio of *Firmicutes* to *Bacteroidetes* can directly affect the fiber metabolism by gut microbiota, consequently changing the concentration of circulating short-chain fatty acids (SCFAs). Production of various SCFAs meditated by gut microbiota has been shown to be important for host systemic immunity. SCFAs, especially butyrate, have exerted broad anti- inflammatory activities (34–36). It will be very interesting to explore the changes of metabolite production after alteration of the ratio of *Firmicutes* to *Bacteroidetes* in further studies on AAD.

## Data Availability Statement

The original contributions presented in the study are included in the article/**Supplementary Material**. Further inquiries can be directed to the corresponding authors.

## Ethics Statement

The studies involving human participants were reviewed and approved by The Affiliated Hospital of Qingdao University Review Board. The patients/participants provided their written informed consent to participate in this study. The animal study was reviewed and approved by The Animal Care Committee of Qingdao University.

## Author Contributions

WP designed and performed most of the experiments. AL, MC, and LH provided help in some of the key experiments. YJ and JZ provided suggestions for the study. ZL and DW supervised the study, analyzed and interpreted data, and wrote the manuscript. All authors contributed to the article and approved the submitted version.

## Funding

This work was supported by the National Natural Science Foundation of China (81700889, 81701381 and 81670908).

## Conflict of Interest

The authors declare that the research was conducted in the absence of any commercial or financial relationships that could be construed as a potential conflict of interest.

## Supplementary Material

Supplementary Figure 1 Mast cell infiltration assessed on Toluidine Bluestained nasal mucosa and lung. **(A)** Mast cells were round or oval, with dark blue nucleus and purplish red or dark purple cytoplasm. Original magnification was ×200. **(B)** Number of mast cells in the nasal mucosa and lung. Bar graphs represent mean ± SD. **P* < 0.05, ***P* < 0.01.

Supplementary Figure 2 Eosinophil inflammation assessed on hematoxylin and eosin (HE) stained nasal mucosa and lung. **(A)** The eosinophils were round and the eosinophilic granules in cytoplasm were brick red or bright red. Original magnification was ×200. **(B)** Number of eosinophils in the nasal mucosa and inflammation score of lung. Bar graphs represent mean ± SD. ***P* < 0.01.

Supplementary Figure 3
*B. thetaiotaomicron* induced ICOS expression on Tregs and amplification of IL-10-expressing CD4^+^Foxp3^+^ Tregs in spleen. Representative scatter plots and ration of the fraction of CD4^+^Foxp3^+^ cells **(A)**, CD4^+^ICOS^+^T cells **(B)**, and CD4^+^ICOS^+^Foxp3^+^ regulatory T cells **(C)**. Representative histogram showing expression of IL-10 in CD4^+^Foxp3^+^ Tregs **(D)**. Bar graphs represent mean ± SD. n = 6, **P* < 0.05, ***P* < 0.01.

Supplementary Figure 4
*B. thetaiotaomicron* induced ICOS expression on Tregs and amplification of IL-10-expressing CD4^+^Foxp3^+^ Tregs in mesenteric LNs. Representative scatter plots and ration of the fraction of CD4^+^Foxp3^+^ cells **(A)**, CD4^+^ICOS^+^T cells **(B)** and CD4^+^ICOS^+^Foxp3^+^ regulatory T cells **(C)**. Representative histogram showing expression of IL-10 in CD4^+^Foxp3^+^ Tregs **(D)**. Bar graphs represent mean ± SD. n = 6, **P* < 0.05, ***P* < 0.01.
