# Supplementary figures and images for: Bacteroides thetaiotaomicron Ameliorates Experimental Allergic Airway Inflammation via Activation of ICOS+Tregs and Inhibition of Th2 Response
Source: Front Immunol. 2021 Mar 17;12:620943. doi: 10.3389/fimmu.2021.620943 (PMC8010693; doi:10.3389/fimmu.2021.620943)

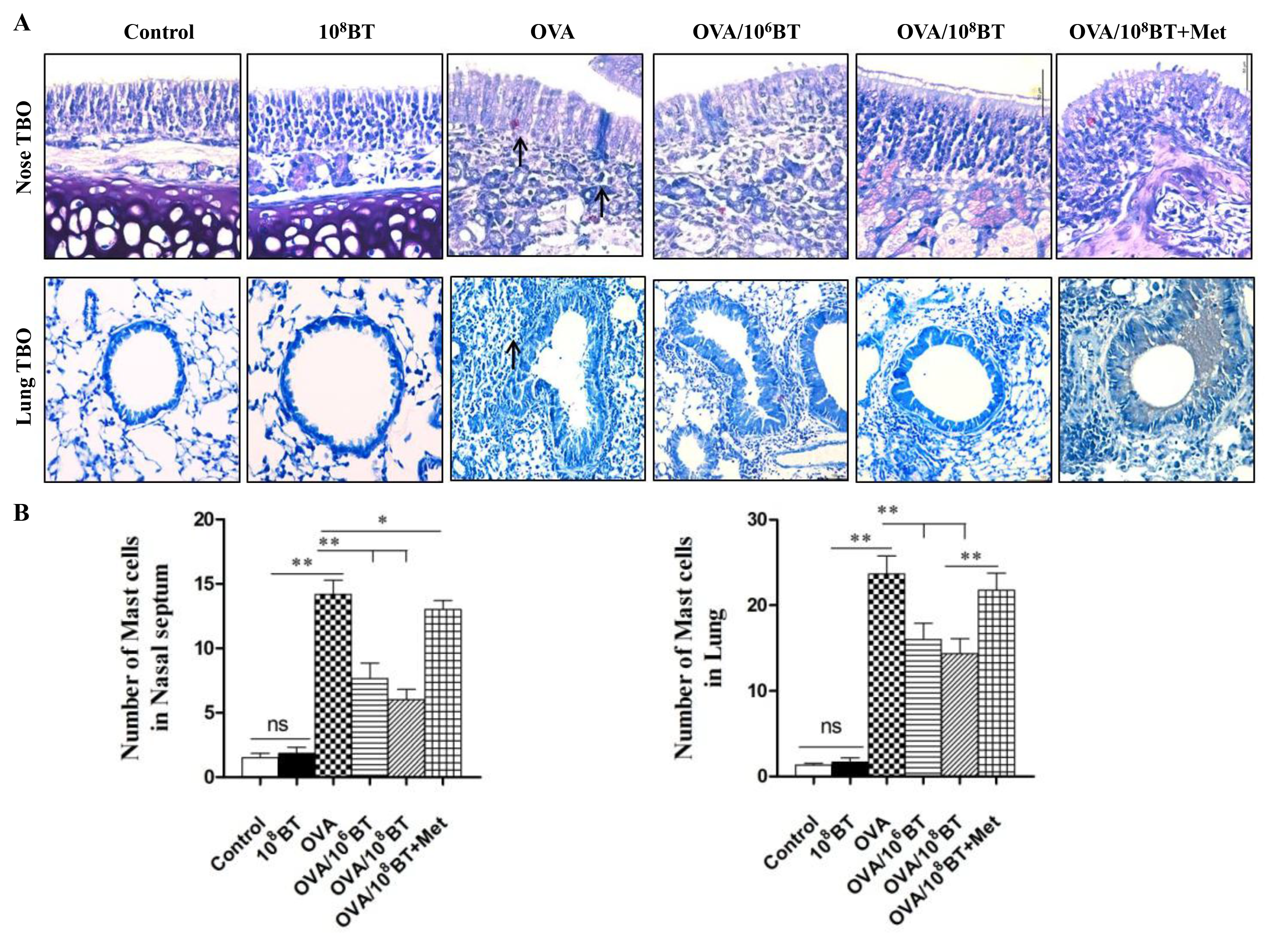

Supplement: Supplementary Figure 1 — Mast cell infiltration assessed on Toluidine Bluestained nasal mucosa and lung. (A) Mast cells were round or oval, with dark blue nucleus and purplish red or dark purple cytoplasm. Original magnification was ×200. (B) Number of mast cells in the nasal mucosa and lung. Bar graphs represent mean ± SD. *P < 0.05, **P < 0.01. [file Image_1.tif]

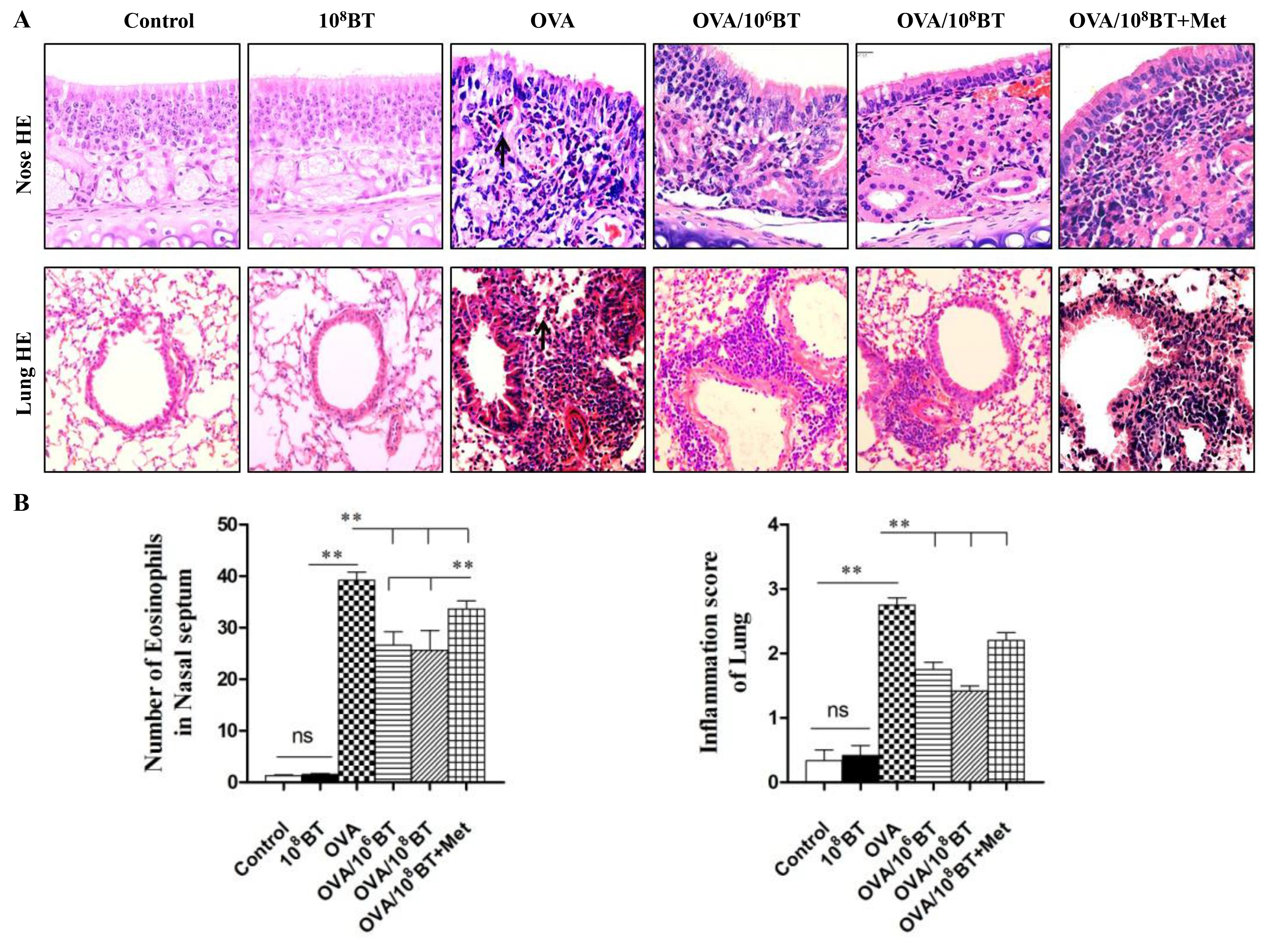

Supplement: Supplementary Figure 2 — Eosinophil inflammation assessed on hematoxylin and eosin (HE) stained nasal mucosa and lung. (A) The eosinophils were round and the eosinophilic granules in cytoplasm were brick red or bright red. Original magnification was ×200. (B) Number of eosinophils in the nasal mucosa and inflammation score of lung. Bar graphs represent mean ± SD. **P < 0.01. [file Image_2.tif]

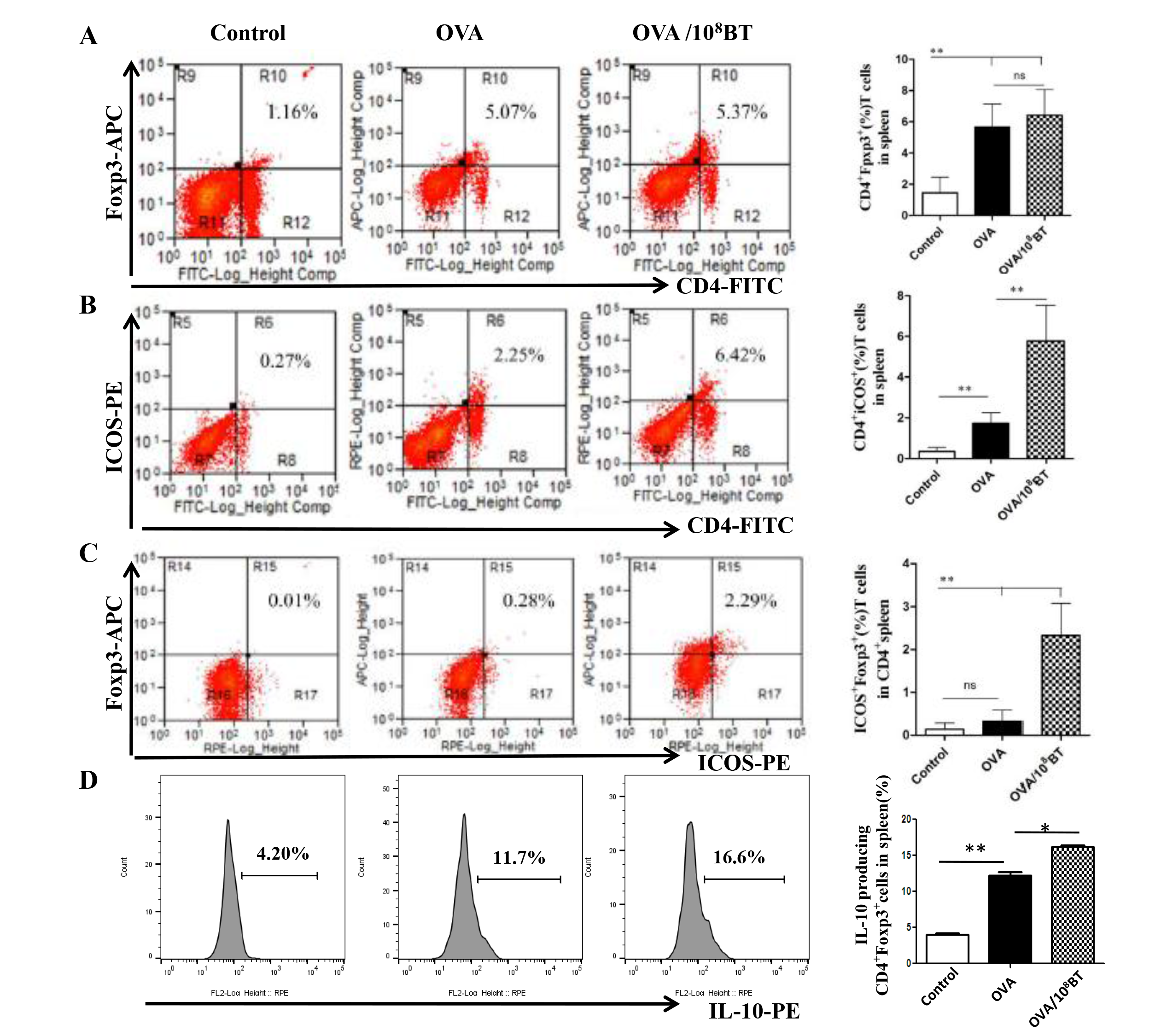

Supplement: Supplementary Figure 3 — B. thetaiotaomicron induced ICOS expression on Tregs and amplification of IL-10-expressing CD4+Foxp3+ Tregs in spleen. Representative scatter plots and ration of the fraction of CD4+Foxp3+ cells (A), CD4+ICOS+T cells (B), and CD4+ICOS+Foxp3+ regulatory T cells (C). Representative histogram showing expression of IL-10 in CD4+Foxp3+ Tregs (D). Bar graphs represent mean ± SD. n = 6, *P < 0.05, **P < 0.01. [file Image_3.tif]

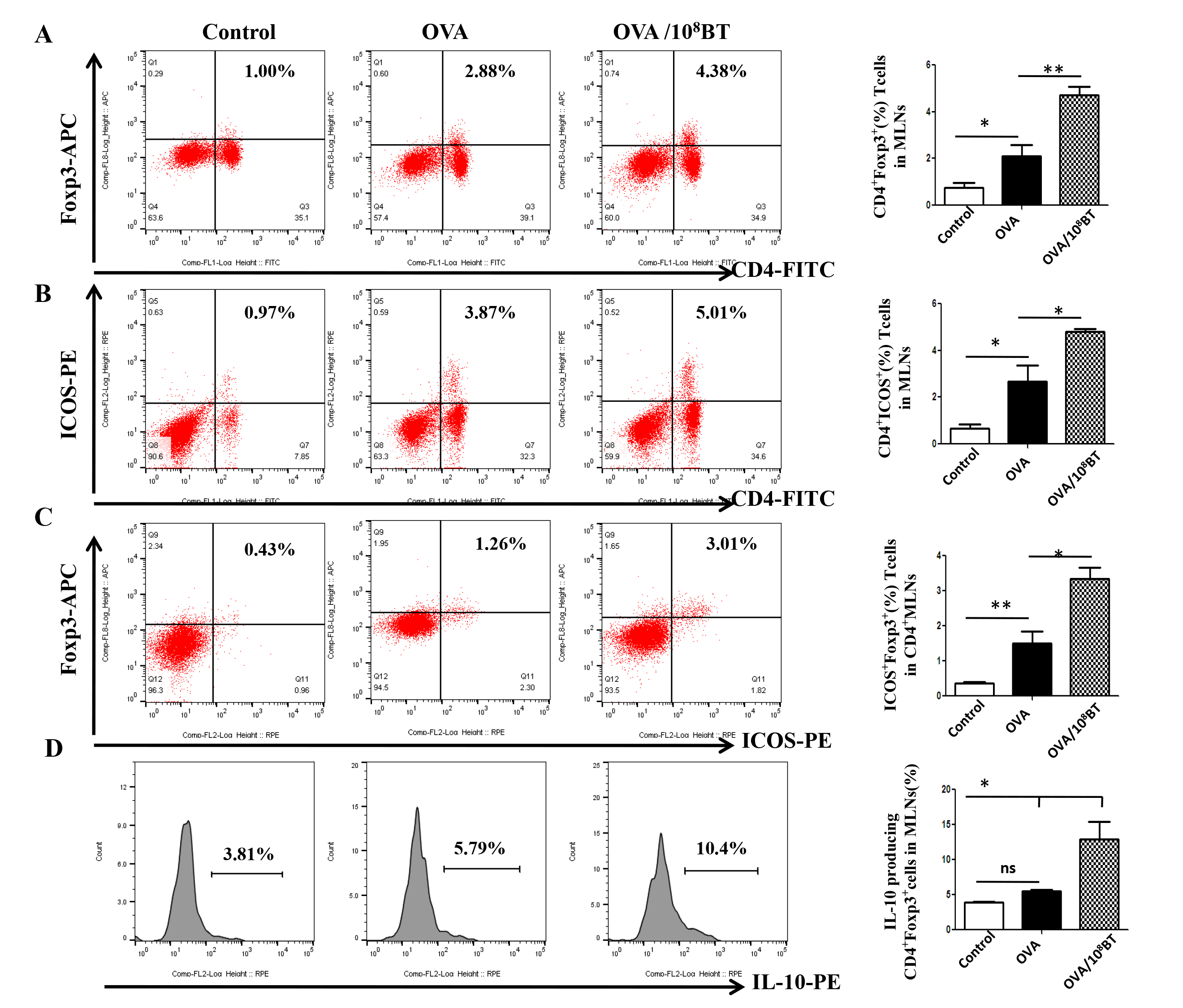

Supplement: Supplementary Figure 4 — B. thetaiotaomicron induced ICOS expression on Tregs and amplification of IL-10-expressing CD4+Foxp3+ Tregs in mesenteric LNs. Representative scatter plots and ration of the fraction of CD4+Foxp3+ cells (A), CD4+ICOS+T cells (B) and CD4+ICOS+Foxp3+ regulatory T cells (C). Representative histogram showing expression of IL-10 in CD4+Foxp3+ Tregs (D). Bar graphs represent mean ± SD. n = 6, *P < 0.05, **P < 0.01. [file Image_4.tif]
